# Fiber optic magnetic field sensor using Co doped ZnO nanorods as cladding

**DOI:** 10.1039/c8ra01803k

**Published:** 2018-05-18

**Authors:** S. Narasimman, L. Balakrishnan, Z. C. Alex

**Affiliations:** School of Electronics Engineering, VIT Vellore 632 014 India zcalex@gmail.com; Department of Physics, Government College of Technology Coimbatore 641 013 India bslv85@gmail.com +91-9944881875

## Abstract

A fiber optic magnetic field sensor is proposed and experimentally demonstrated. Pristine and Co doped ZnO nanorods of different Co concentrations (5, 10, 15 and 20 at%) were synthesized using a hydrothermal method. The synthesized nanorods were subjected to various characterization methods like X-ray diffraction (XRD), optical absorption, scanning electron microscopy, energy dispersive X-ray spectroscopy, Fourier transform infrared spectroscopy, vibrating sample magnetometry and X-ray photoelectron spectroscopy (XPS). XRD and XPS analysis confirms that the Co ions were successfully incorporated into the Zn site of the wurtzite ZnO lattice without altering the structure. The pristine and Co doped ZnO nanorods showed remarkable changes in the *M*–*H* loop where the diamagnetic behavior of ZnO changes to paramagnetic when doped with Co. The sensor structure is composed of cladding modified fiber coated with Co doped ZnO nanorods as a sensing material. The modified cladding is proportionally sensitive to the ambient magnetic field because of the magneto-optic effect. Experimental results revealed that the sensor has an operating magnetic field range from 17 mT to 180 mT and shows a maximum sensitivity of ∼18% for 15 at% Co doped ZnO nanorods. The proposed magnetic field sensor would be attractive due to its low cost fabrication, simplicity of the sensor head preparation, high sensitivity and reproducibility.

## Abstract 2

A fiber optic magnetic field sensor based on Co doped ZnO nanorods is proposed and demonstrated. The sensor has an operating magnetic field range of 17 mT to 180 mT and shows a maximum sensitivity of ∼18% for 15 at% Co doped ZnO nanorods.

## Graphical abstract

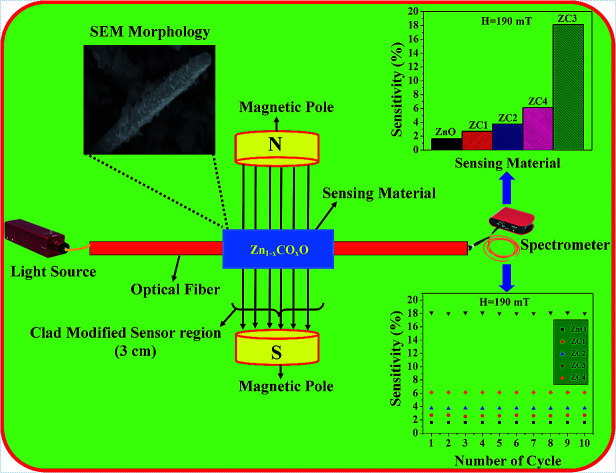

## 1. Introduction

Nanostructured metal oxides have acquired a significant role in the scientific world due to their efficient technological applications in the field of solar cells, optoelectronic devices, gas sensors and spintronics.^1–5^ ZnO is a unique wide band gap (3.37 eV) semiconductor with a large exciton binding energy (60 meV) and good chemical stability.^6^ The inception of impurities into the ZnO host is an efficient way to produce novel properties in ZnO. Diluting non-magnetic ZnO with transition metal dopants such as Mn^2+^, Co^2+^, Ni^2+^ and Fe^2+^ could deliver different magnetic properties. This new genre of semiconductor is entitled as dilute magnetic semiconductors.^7,8^

In the last few decades, many researchers have curiously investigated the impact of doping ZnO nanostructures with TM ions. Among the various TM ions, Co has its own importance in that (i) it is more reconcilable with Zn while doping, (ii) it can amend the morphology and optical properties of ZnO nanostructures, (iii) easily soluble in ZnO nanostructures, (iv) it has a strong magnetic moment (*μ*_Co_ = 1.8 *μ*_B_) compared with other transition metals and (v) the ionic radius of Zn^2+^ (0.60 Å) is nearly same as Co^2+^ (0.58 Å).^9^ Many research groups have synthesized Co doped ZnO nanostructures and assessed their performance on doping. For instance, Sharma *et al.* have synthesized Co : ZnO nanoparticles using a co-precipitation method and showed their ferromagnetism at room temperature.^10^ Xu *et al.* have synthesized Co doped ZnO nanoflakes using a hydrothermal method and noticed their ferromagnetism at room temperature. However, with increasing Co^2+^ dopant concentration, paramagnetism was exhibited.^11^ Qiu *et al.* have also reported room temperature ferromagnetism (RTFM) in Co : ZnO by water-bubble template process.^12^

In the past few years, several techniques have been ratified to synthesize pristine ZnO and TM doped ZnO nanostructures including co-precipitation,^13^ hydrothermal,^14^ sol–gel,^15^ magnetron sputtering,^16^ ball milling,^17^*etc.* Of these, the hydrothermal method offers various nanostructure morphologies, controlled particle size, high reaction rate, different phase formation, *etc.*^18^ To explore the potential of nanoparticles in the field of magnetic field sensing, various types of sensor such as thin films,^19^ fiber optics,^20^ Hall effect based magnetic field sensors and magnetic switches have been fabricated. Though, fiber optic based magnetic sensors offer numerous advantages such as small size, on-line analysis, remote sensing, high sensitivity, immunity to electromagnetic interference and a capability of working in harsh environments.^21^ In fiber optic sensor, the principle of sensing is based on cladding modification technology, in which the middle part of the cladding was replaced with a magnetic sensing material. The magnetic field sensing material causes the magneto-optic effect such as change in refractive index as a result of an applied external magnetic field.

Hitherto, various kinds of fiber optic based magnetic sensor have been proposed using magnetic fluid (MF) as the cladding with different optical devices such as Fiber Bragg Grating (FBG),^22^ Long Period Fiber Bragg Grating (LPFG),^23^ single-mode–multi-mode–single-mode (SMS) structures,^24^ multi-mode–single-mode–multi-mode (MSM) structures,^25^ micro resonators,^26^ and Fabry–Pérot devices.^27^ However, minor shortcomings like the feeble response of MF to external magnetic fields, low interaction between core mode/lower order cladding mode and MF, structure instability and complicated technology mean that they fail to achieve higher magnetic sensitivity.

To overcome these shortcomings, a fiber optic magnetic field sensor based on Co doped ZnO nanorods is proposed and demonstrated. The impact of doping concentration on structural, optical and magnetic properties is also investigated.

## 2. Materials and methods

### 2.1. Synthesis of pristine and Co doped ZnO nanorods

Pristine ZnO and Zn_1−*x*_Co_*x*_O (*x* = 5, 10, 15 and 20 at%) were synthesized using a hydrothermal method. All the chemicals used in this work were analytical reagent grade and used as received without further purification. Zinc acetate dihydrate (Zn(CH_3_COO)_2_·2H_2_O, 99.99%, Sigma-Aldrich) and cobalt(ii) chloride, (99.99%, Sigma-Aldrich) in suitable weight percentages were used as starting materials. Additionally, sodium hydroxide (NaOH, 99.99%, Alfa Aesar) was used as the precipitating agent and cetyl trimethyl ammonium bromide (CTAB) (C_19_H_42_BrN) was used as a surfactant.

For the synthesis of pristine ZnO nanorods, 50 ml of 0.2 M zinc acetate dihydrate, 50 ml of 0.1 M CTAB and 100 ml of 2 M NaOH aqueous solution were prepared. Initially, zinc acetate dihydrate solution was stirred continuously for 3 h and aqueous NaOH solution was added dropwise to the mixture until the pH value of solution reaches 8.0. Later, the white precipitated solution was stirred for 1 h. Finally, CTAB solution was added into the mixture and stirred vigorously for another 30 min. Then, the final solution was transferred into a 100 ml teflon-lined autoclave and maintained at 170 °C for 72 h. After the reaction was completed, the resultant product was washed several times with distilled water and ethanol alternatively, and dried at 60 °C overnight.

For the synthesis of Zn_1−*x*_Co_*x*_O (*x* = 5, 10, 15 and 20 at%) nanorods, the calculated amount of cobalt(ii) chloride and zinc acetate dihydrate were dissolved in 50 ml of distilled water and stirred for 3 h. Then, aqueous NaOH solution was added drop wise into the above mixture to form a white precipitate with a slight pale pink colour. After that the same procedure that was adopted for the synthesis of pristine ZnO nanorods was followed for the synthesis of Co doped ZnO nanorods.

### 2.2. Sensor head preparation and setup

**Fig. 1:**
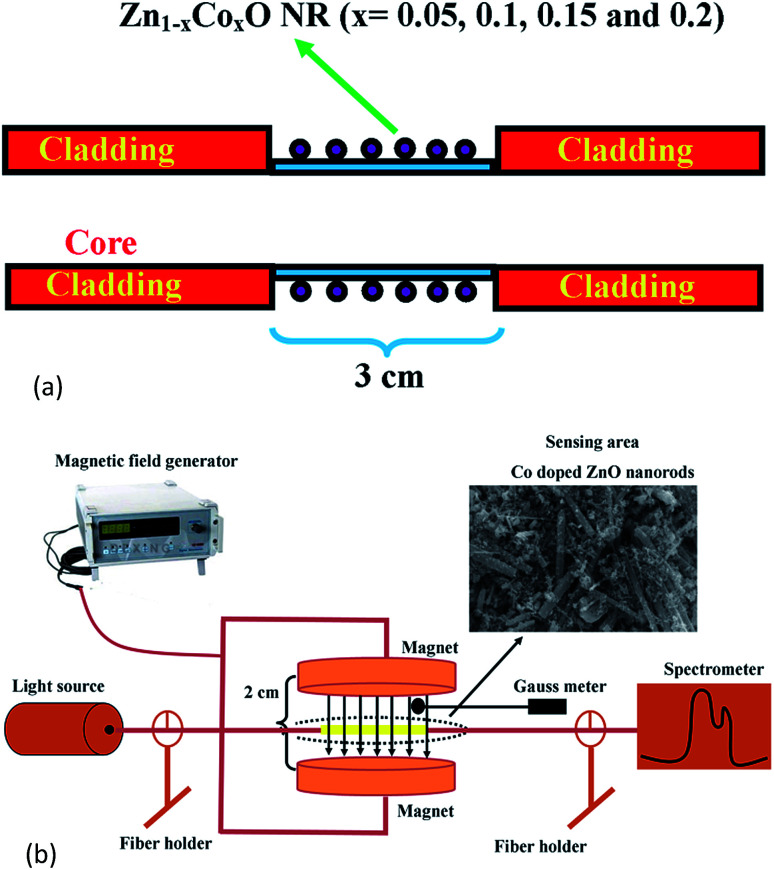
(a) Schematic diagram of the proposed magnetic field sensing structure. (b) Experimental setup for the magnetic field sensor.

Fig. 1(a) shows the schematic diagram of the proposed magnetic field sensor structure. A multi-mode plastic optical fiber of about 42 cm length with 1000 μm diameter and 0.51 numerical aperture was used as a sensor head. A cladding region of about 3 cm in length was mechanically removed at the center portion of the fiber using a chemical etching process followed by fine polishing. The ablated cladding region was chemically etched using acetone followed by polishing with a 1000 grid sheet. Then, the polished surface was cleaned and coated with Co (at% of 5, 10, 15 and 20) doped ZnO nanorods using a dip coating method.

The scheme of the experimental setup used for measuring the magnetic field sensing characteristics of the fabricated sensor is shown in Fig. 1(b). In the sensor setup, a broadband light source (halogen lamp-SLS201/M) with the wavelength ranging from 300 to 2600 nm is coupled at one end of the fiber and the intensity spectrum was recorded with a fiber optic spectrometer (CCS200/M) having a spectral range of 200 to 1000 nm at the other end of the fiber. The sensor head was inserted between the two poles of an electromagnet which generates a static magnetic field around the sensing head. The experiment was conducted at ambient temperature (28 °C).

## 3. Results and discussion

### 3.1. Structural analysis

5, 10, 15 and 20 at% Co doped ZnO nanorods are represented as ZC1, ZC2, ZC3 and ZC4 respectively.

**Fig. 2:**
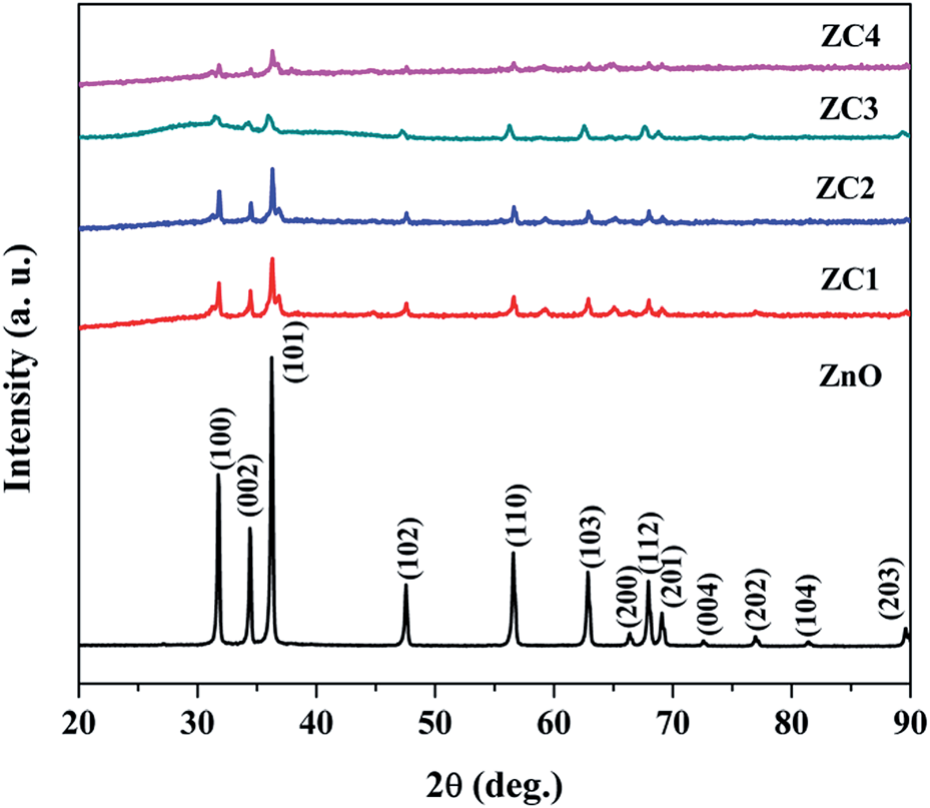
XRD pattern of pristine ZnO and Zn_1−*x*_Co_*x*_O nanorods.

XRD patterns of pristine ZnO and Zn_1−*x*_Co_*x*_O (*x* = 0.05, 0.1, 0.15 and 0.2) nanorods are shown in Fig. 2. All the diffraction peaks from the XRD patterns clearly manifest that the Zn_1−*x*_Co_*x*_O nanorods addresses the hexagonal wurtzite structure and are very well matched with standard JCPDS card no: 36-1451. No other secondary phases of Co clusters were found. The non-existence of impurity peaks reveal that Co ions were successfully incorporated into the ZnO lattice. The decrease in diffraction peak intensity is due to an increase in the concentration of impurities.^28^ The average crystallite size and strain of the nanorods are calculated from the Williamson and Hall (W–H) plot.^29^

The average crystallite size and strain of the nanoparticles are shown in Table 1. It was found that due to the incorporation of impurities, the crystallite size decreases.

**Table 1:** Crystallite size and microstrain of pristine ZnO and Zn_1−*x*_Co_*x*_O nanorods

Sample	Crystallite size (*D*) (nm)	Microstrain (*ε*)
ZnO	65	0.00070
ZC1	63	0.00073
ZC2	57	0.00076
ZC3	53	0.00082
ZC4	49	0.00085

### 3.2. Morphological and elemental analysis

**Fig. 3:**
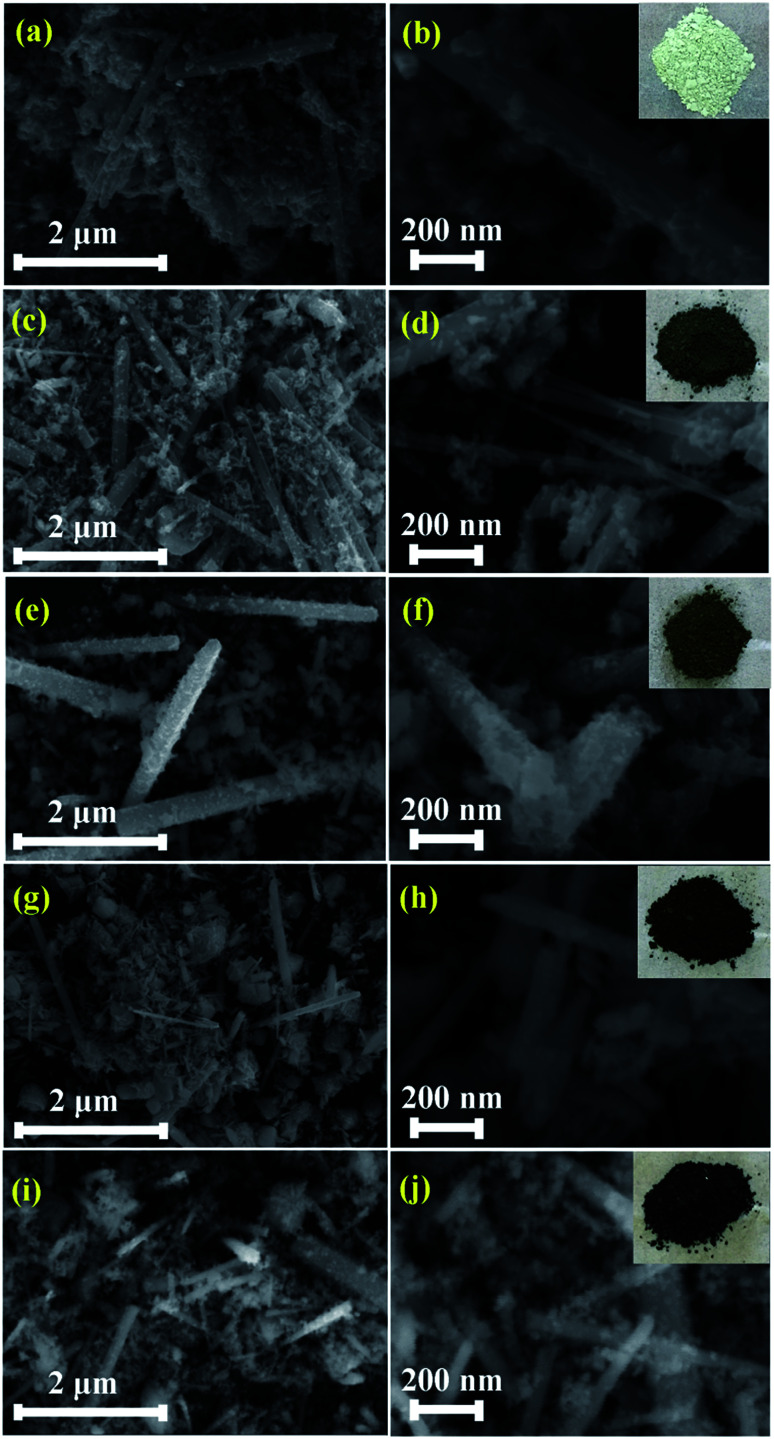
SEM images of pristine ZnO and Zn_1−*x*_Co_*x*_O nanorods.

**Fig. 4:**
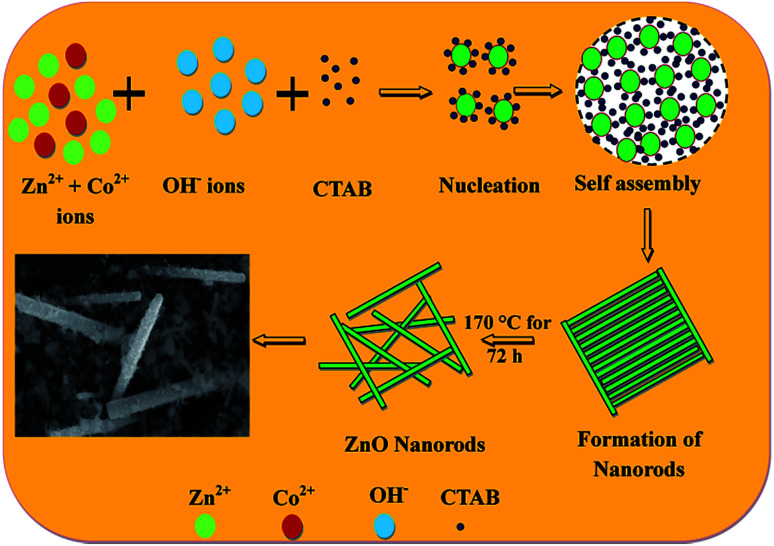
Schematic formation mechanism of Zn_1−*x*_Co_*x*_O nanorods.

Fig. 3(a–j) shows the morphologies of pristine and Zn_1−*x*_Co_*x*_O nanoparticles at different magnifications. It is seen that the synthesized nanopowder shows a rod like morphology. In order to analyse the complete morphology information of the ZnO nanorods, a plausible formation mechanism for the ZnO nanorods was proposed, as schematically illustrated in Fig. 4.

The formation mechanism of Co doped ZnO nanorods can be invoked *via* the following chemical reactions:

Decomposition of Zn(Ac)_2_:

1 Zn(CH_3_COO)_2_·2H_2_O + 2NaOH → Zn(OH)_2_ + 2CH_3_COONa + 2H_2_O

Production of pristine ZnO nanoparticles:

2 Zn(OH)_2_ ↔ Zn^2+^ + 2OH^−^

3 Zn(OH)_2_ + 2OH^−^ → [Zn(OH)_4_^2−^]

4 [Zn(OH)_4_^2−^] → ZnO + OH^−^ + H_2_O

Decomposition of CoCl_2_:

5 CoCl_2_ + 2NaOH → Co(OH)_2_ + 2NaCl

6 Co(OH)_2_ ↔ Co^2+^ + 2OH^−^

Production of Co doped ZnO nanoparticles:

7 Zn_1−*x*_(OH)_2_ + Co_*x*_(OH)_2_ → Zn_1−*x*_Co_*x*_O + H_2_O

Reaction (1) and (5) shows the decomposition of Zn(Ac)_2_ and CoCl_2_ in an ambient environment producing Zn(OH)_2_ and Co(OH)_2_, respectively. If the pH value in aqueous solution is about 11, where Zn(OH)_2_ is the main chemical compound, during the hydrothermal method, a part of the Zn(OH)_2_ colloid species dissolves into Zn^2+^ and OH^−^ according to reaction (2). When the concentration of Zn^2+^ and OH^−^ reaches the super saturation degree of ZnO, then ZnO nuclei will form according to reaction (3) and (4). Reaction (6) indicates the dissolution of Co(OH)_2_ in water to produce Co^2+^ ions that will be incorporated into the ZnO lattice. Reaction (7) is the final step of the growth process to accomplish Co doped ZnO nanoparticles.^30^ To obtain the consistent nanoparticle size, the headway approach to synthesize Co doped ZnO nanoparticles using a hydrothermal method is closely related to a new understanding of the formation mechanism by the introduction of the surfactant CTAB. CTAB is a cationic surfactant having small hydrophilic head and a hydrophobic tail. The element Zn was obtained in [Zn(OH)_4_]^2−^ as a negatively charged tetrahedrons that were formed according to reaction (3), whereas CTA^+^ was positively charged with a tetrahedral head. At a higher reaction temperature (∼170 °C), the cationic charge of CTA^+^ and anionic charge of [Zn(OH)_4_]^2−^ were formed primarily by electrostatic interactions (reaction (4)). The CTAB could accelerate the ionization of [Zn(OH)_4_]^2−^ as it is a strong acid–weak-base salt.^31^ It was assumed that the CTAB was aggregated in between the ZnO crystallites during hydrothermal crystallization and after washing thrice with ethanol, the rod like structure of ZnO was formed.

**Fig. 5:**
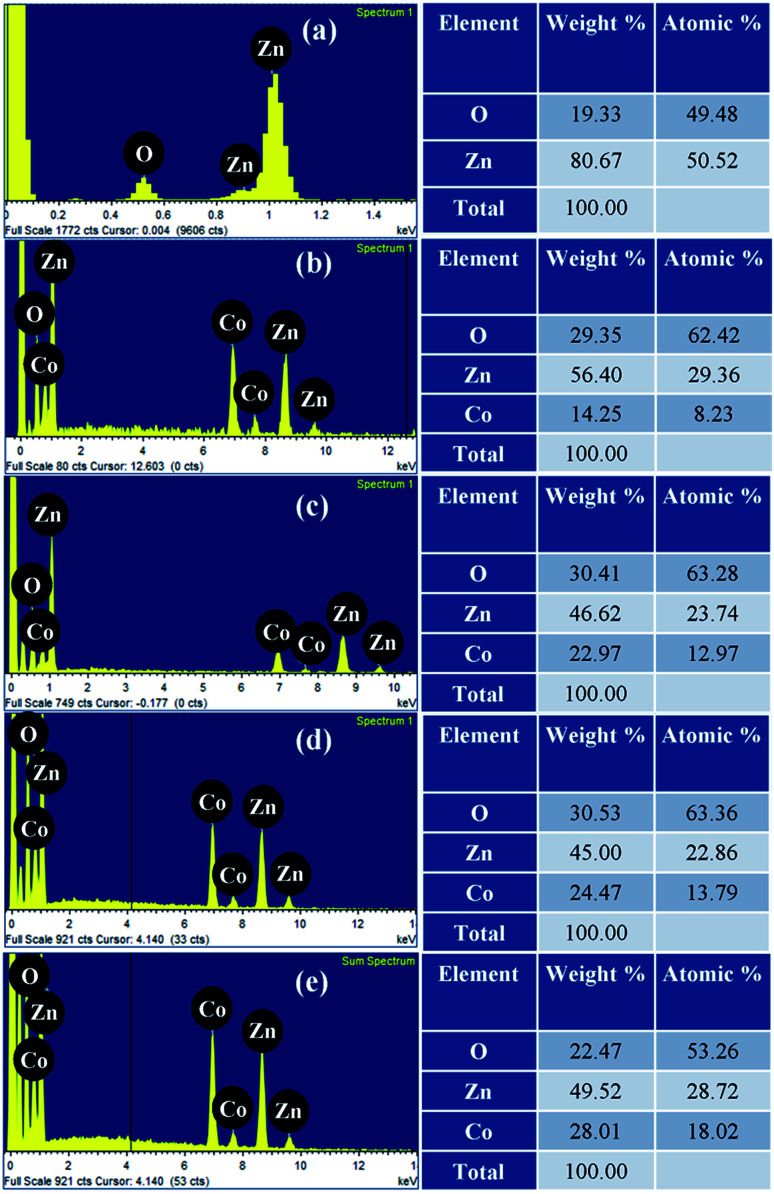
EDS spectra of pristine ZnO and Zn_1−*x*_Co_*x*_O nanorods.

**Fig. 6:**
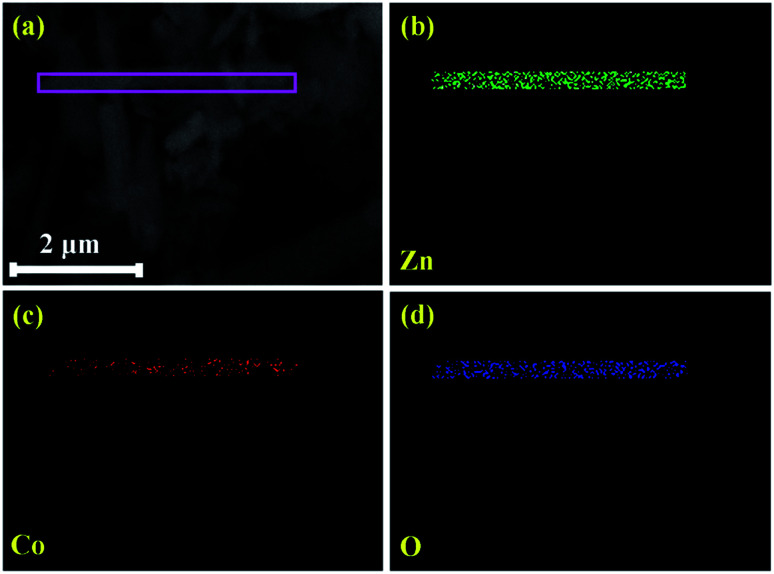
Elemental mapping of ZC3 nanorods.

The elemental composition of pristine and Zn_1−*x*_Co_*x*_O nanorods were investigated using EDS spectra and shown in Fig. 5(a–e). The spectra affirm the presence of Zn and O in ZnO, likewise, Zn, O and Co in Co doped ZnO nanorods. Fig. 6 shows the elemental mapping of Zn, O and Co on a single Co doped ZnO nanorod. The mapping results indicated that Zn, O and Co atoms are uniformly distributed on the single nanorod.

### 3.3. FTIR analysis

**Fig. 7:**
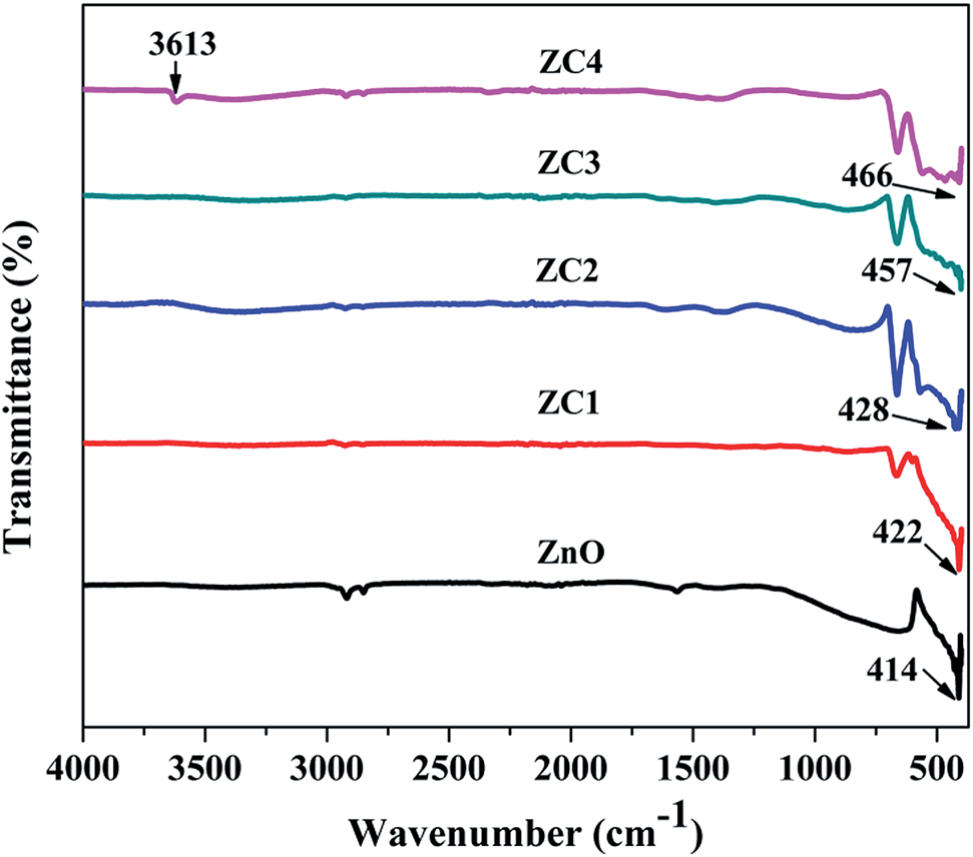
FTIR spectrum of pristine and Co doped ZnO nanorods.

Fig. 7 shows the vibrational frequencies of pristine and Co doped ZnO nanorods. The peak obtained between 3420–3650 cm^−1^ corresponds to the stretching vibration of O–H.^32^ The stretching vibrations of Zn–O are observed at 414, 422, 428, 457 and 466 cm^−1^ for pristine and Co doped ZnO nanorods.^33^

Upon Co doping, the IR peak shifts consistently from 414 cm^−1^ to 466 cm^−1^ and this shift is attributed to the incorporation of Co^2+^ ions in the ZnO lattice.^34^

### 3.4. Optical absorption studies

**Fig. 8:**
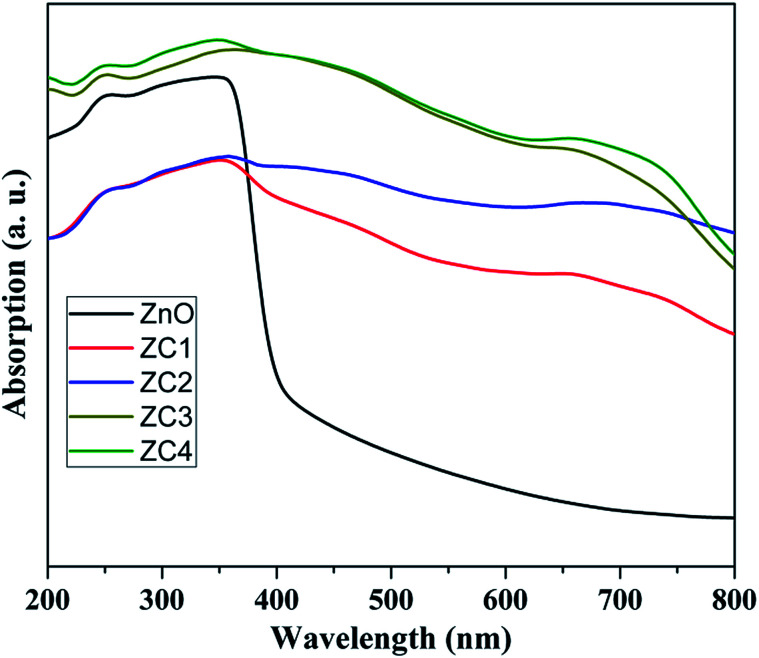
Absorption spectrum of pristine and Co doped ZnO nanorods.

Fig. 8 shows the optical absorption spectra of pristine and Co doped ZnO nanorods recorded in DRS mode. The absorption edge of the Co doped ZnO depicts significant red shift compared with pristine ZnO. It is attributed that the Co doped ZnO nanorods showed absorption from UV to visible region due to the d–d transitions.^35^ This affirms the incorporation of Co^2+^ ions into the ZnO lattice, rather than forming as cobalt oxide (CoO) or Co metal.

### 3.5. VSM analysis

**Fig. 9:**
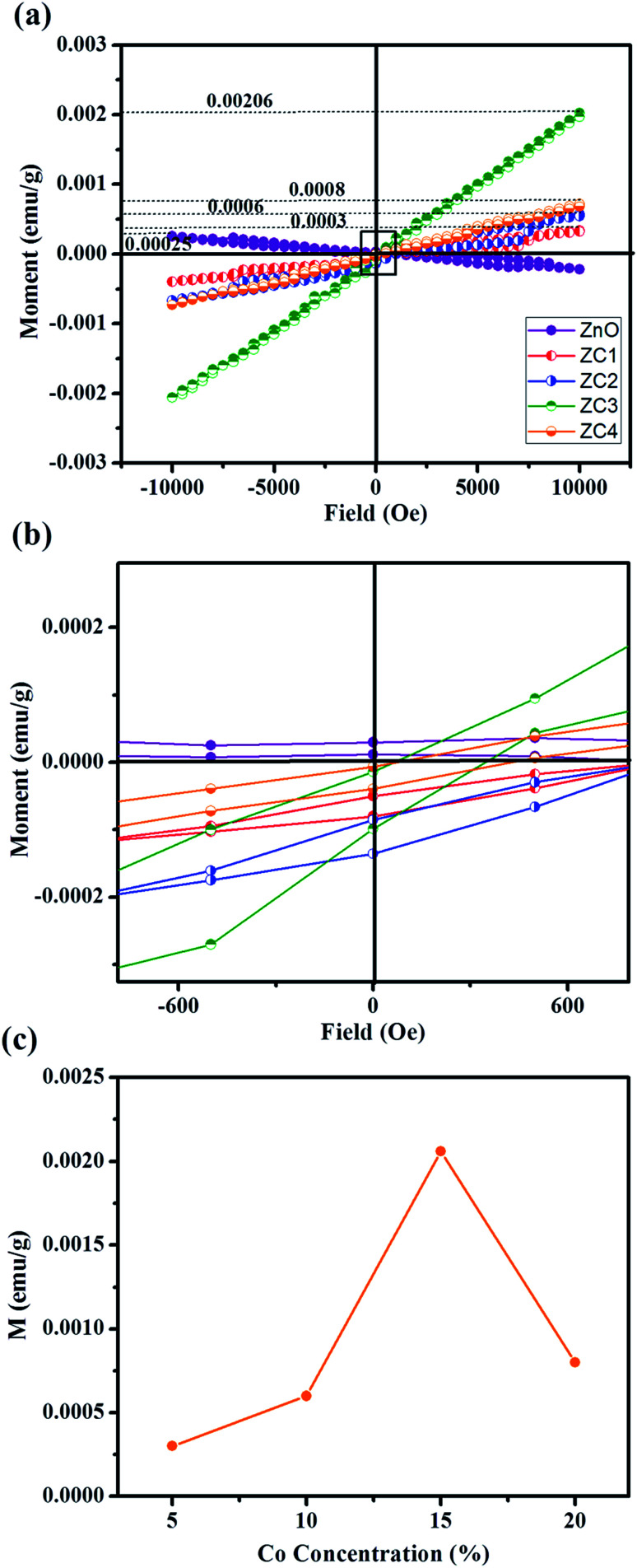
(a) Magnetic field dependent magnetization curve of pristine ZnO and Zn_1−*x*_Co_*x*_O nanorods at 300 K. (b) Zoomed magnetization plot and (c) variation of magnetization *M* with Co concentration.

Fig. 9 shows magnetization *versus* magnetic field (*M*–*H*) measurements with maximum applied field ±10 kOe at room temperature (300 K). All the Co doped ZnO nanorods revealed paramagnetic behaviour excluding pristine ZnO (diamagnetic behaviour). The diamagnetic behaviour of pristine ZnO is ascribed to the presence of paired electrons in its d orbital. The 5% and 10% Co doped ZnO nanorods (ZC1 and ZC2) deduces weak ferromagnetism (Fig. 9(b)). It is suggested that a few of the doped Co^2+^ cations occupy the next nearest lattice sites. The nearest Co–Co pairs couple in an antiferromagnetic way and suppress the magnetization.^36–39^ Thus, weak ferromagnetism was observed in the ZC1 and ZC2 samples. When the level of Co doping increases to 20%, the sample demonstrates linear magnetization curves with the absence of a hysteresis loop within the applied field (ZC3 and ZC4), which can be concluded as good paramagnetism. As Co dopant concentration increases, more and more nearest Co–Co pairs exhibit larger antiferromagnetic interactions, which leads to the vanishing of hysteresis in the ZC3 and ZC4 samples. This implies that higher Co doping concentrations in ZnO lead to paramagnetism.^40^ Similar paramagnetic behaviour with the same composition has been reported by many research groups.^41–45^ However, for higher magnetizing fields the magnetization is found to increase with increasing Co concentration (Fig. 9(c)).

### 3.6. XPS analysis

**Fig. 10:**
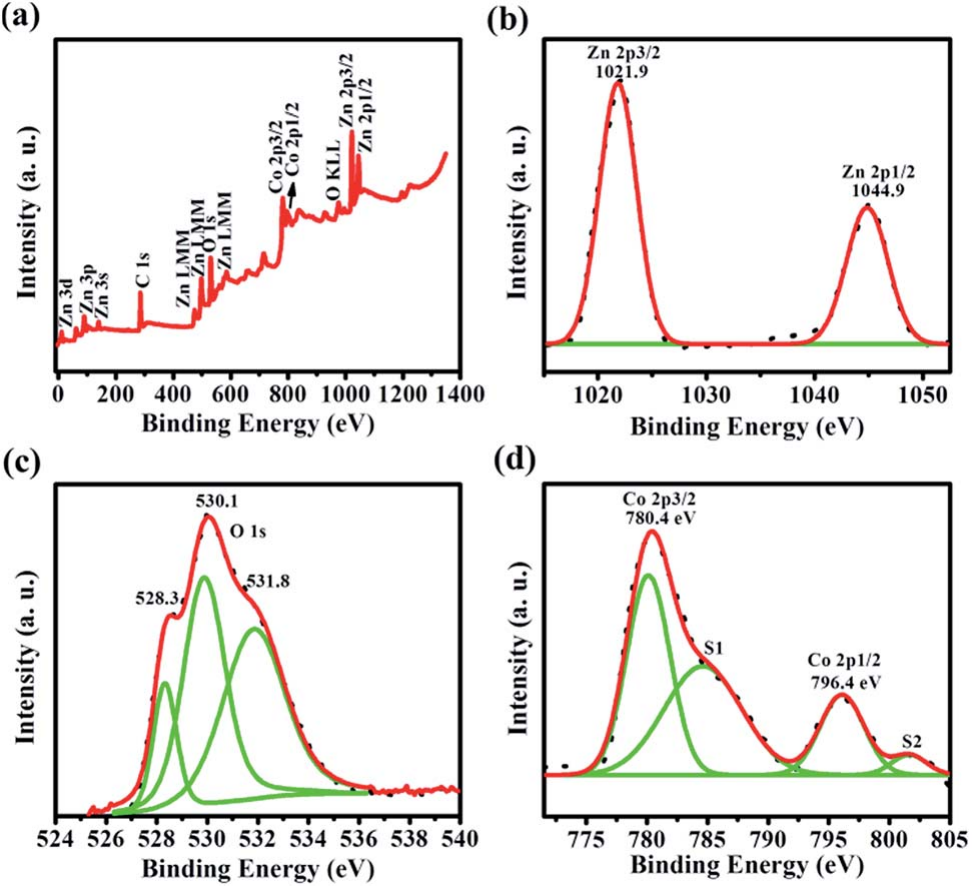
(a) XPS survey spectrum of Co doped ZnO nanorods (*x* = 0.15). (b–d) High resolution spectra of Zn, O and Co, respectively.

Furthermore, to confirm the presence of elements and its chemical bonding states in synthesized Co doped ZnO nanorods (*x* = 0.15), XPS analysis has been carried out. Fig. 10(a) depicts the full range survey spectrum of Co doped ZnO nanorods (*x* = 0.15), which reveals the presence of characteristic peaks of Zn, O and Co in the synthesized Co doped ZnO nanorods. The selected data was corrected with C 1s carbon contamination peak (284.6 eV). Fig. 10(b) shows the high resolution spectra of the Zn 2p energy state. The core level binding energy of Zn 2p_3/2_ and Zn 2p_1/2_ was observed at 1021.9 eV and 1044.9 eV, respectively. The energy difference between these two peaks (∼23 eV) confirms that Zn exists primarily in the Zn^2+^ chemical state.^46^Fig. 10(c) depicts three distinct characteristic peaks of the O 1s energy state observed at 528.3 eV, 530.1 eV and 531.8 eV, which are ascribed to the formation of three different O species in the synthesized nanorods. The lower binding energy implies that lattice oxygen in hexagonal wurtzite structure is surrounded by zinc and cobalt ions. The medium binding energy affirms the presence of oxygen vacancies in the ZnO matrix. The higher binding energy attributes the formation of adsorbed oxygen (O^2−^) on the surface of the ZnO nanorods.^47^

Fig. 10(d) shows the Co 2p energy state high resolution spectra. The two characteristic peaks of Co 2p_3/2_ and Co 2p_1/2_, located at 780.4 eV and 796.4 eV, respectively, have been observed. In the high resolution spectra of Co 2p, the energy difference between Co 2p_3/2_ and Co 2p_1/2_ energy states is ∼16 eV, clearly showing that Co was successfully incorporated as a divalent ion. This is very well matched with previous literature reports.^48–50^ In addition to this, two shake-up satellite peaks (S1 and S2) were observed at 785 eV and 796.4 eV along with the main characteristic peaks of the Co 2p energy state. Thus, XPS analysis clearly reveals that Co^2+^ was successfully incorporated into the ZnO lattice by substituting Zn^2+^ without any additional impurities or phases.

### 3.7. Magnetic field sensing analysis

#### 3.7.1. Sensing mechanism

**Fig. 11:**
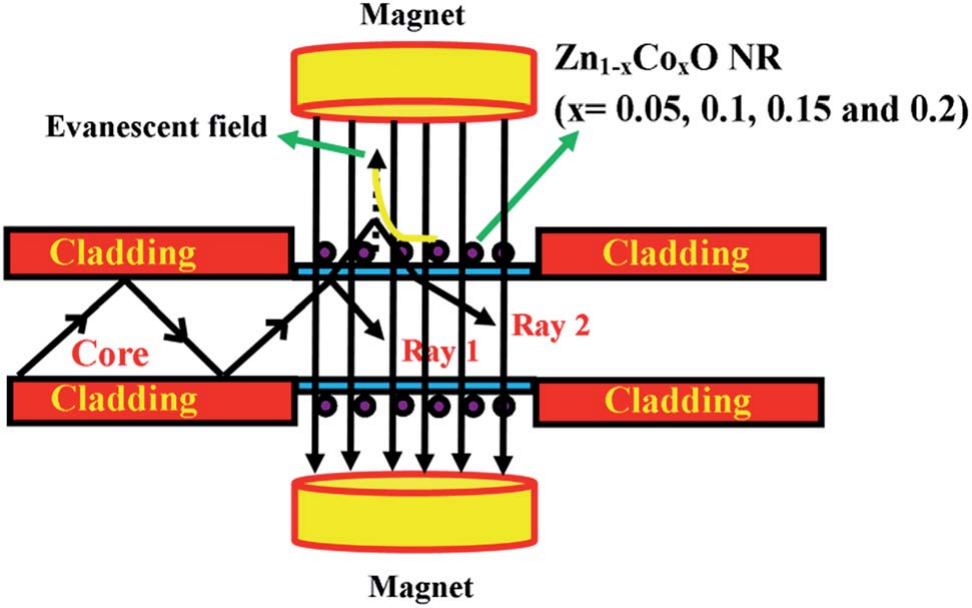
Scheme of the magnetic field sensing mechanism in the proposed magnetic field sensor.

Fig. 11 shows the magnetic field sensing mechanism in the proposed fiber optic sensor. In the clad modified fiber optic sensor, the output light spectral variation with applied magnetic field leads the evanescent wave absorption in the modified cladding due to the change in the refractive index. When light travels with total internal reflection at the interface of the core and modified cladding, not all of the light intensity is reflected back but a part of it penetrates into the cladding material and its intensity decays exponentially away from the interface. This phenomenon is called an evanescent field.^51^ When an external magnetic field was subjected transversely to the direction of incident light and interacts with the evanescent field, the transmitted light spectrum and the output light intensity of the sensor varies. The reason for the change in intensity variation for the cladding modified fiber in an external magnetic field is based on the following relation,^52–54^8
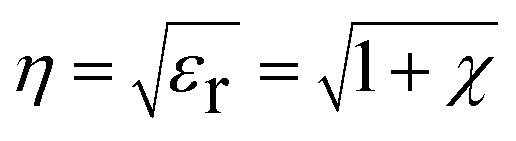


From the equation, *ε*_r_ is the dielectric constant and *χ* is the electric susceptibility. When an external magnetic field is applied perpendicularly to the direction of the propagated light, then9
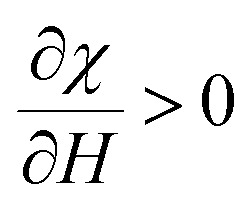


So the refractive index of Co doped ZnO nanorods will increase with increasing in magnetic field strength. In the present work, a part of the cladding (*n*_clad_ = 1.402) was replaced with the synthesized pristine and Co doped ZnO nanorods (*n*_ZnO_ = 1.91) which contributes to a certain amount of decreased attenuation in the guided signal depending on the absorbance of the cladding.

Hence, the change in refractive index and the absorbance of the cladding should affect the total internal reflection and evanescent field respectively. This is reflected in the change in intensity of the signal which is guided along the fiber.^55^ The proposed fiber optic sensor works in a leaky mode condition as the refractive index of the modified cladding (*n*_ZnO_ = 1.91) is higher than that of the core (*n*_core_ = 1.492).

#### 3.7.2. Spectral analysis

**Fig. 12:**
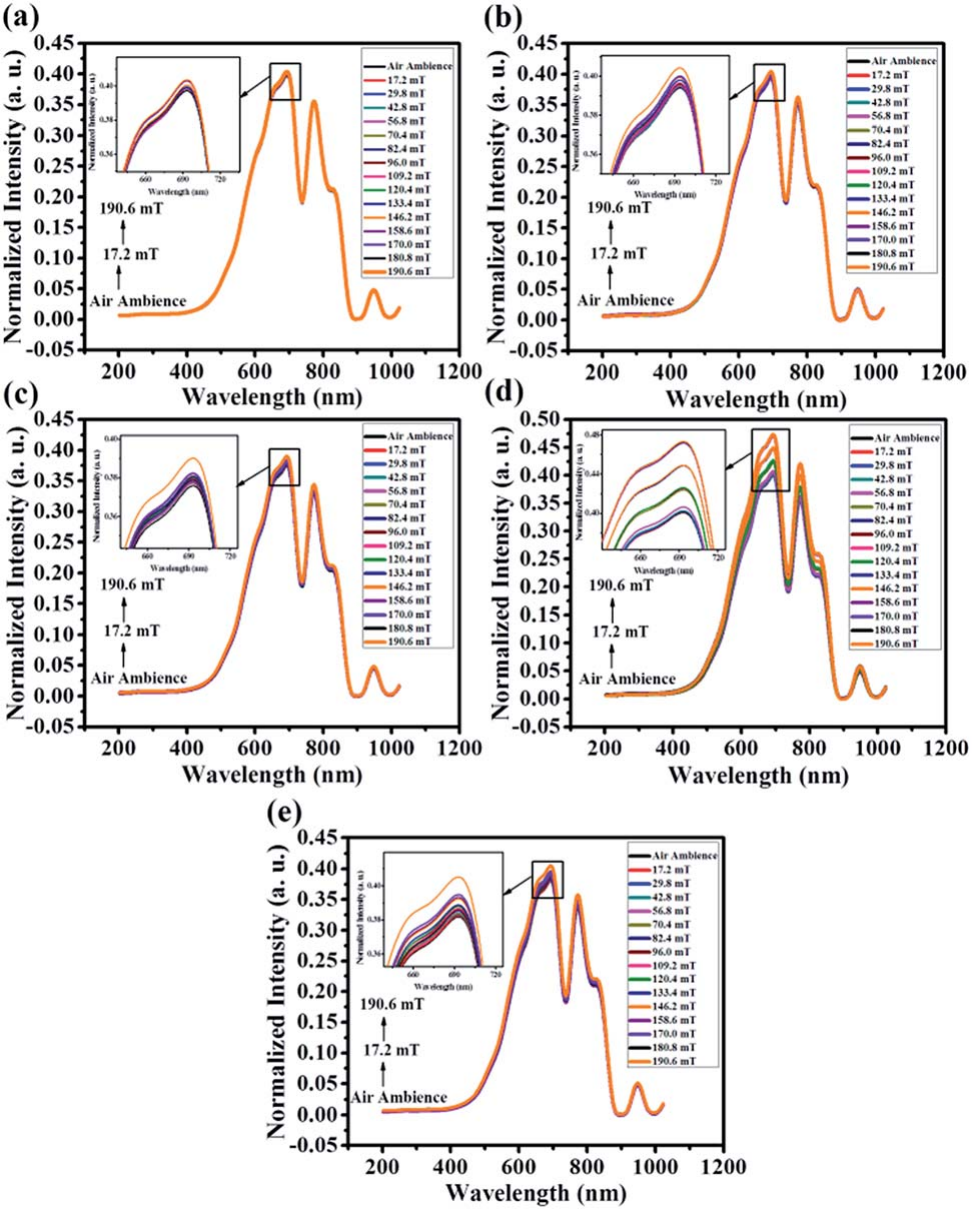
Spectral response of the proposed sensor with different magnetic field strength ranging from 17.2 mT to 190.6 mT (a) ZnO, (b) ZC1, (c) ZC2, (d) ZC3 and (e) ZC4.

Fig. 12(a–e) shows the magnetic field sensing characteristics of pristine and Co doped ZnO nanorods with the magnetic field ranging from 17.2 mT to 190.6 mT. The spectra exhibit three peaks around 693, 772 and 946 nm which are characteristic of the optical fiber used. These spectra suggest that the spectra only undergo intensity variation for different magnetic field strengths. It is clearly seen from the figure that the spectral intensity increases monotonically with increasing applied magnetic field strength. This is due to the decrease in evanescent wave absorption with increasing magnetic field. The light intensity in the absence of a magnetic field is taken as a reference.

#### 3.7.3. Sensitivity analysis

The sensitivity of the proposed magnetic field sensor is calculated using the following relation,^56^10
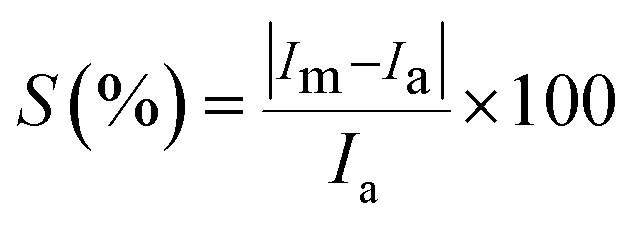
where *I*_a_ is the intensity in the absence of a magnetic field and *I*_m_ is the intensity in the presence of a magnetic field.

**Fig. 13:**
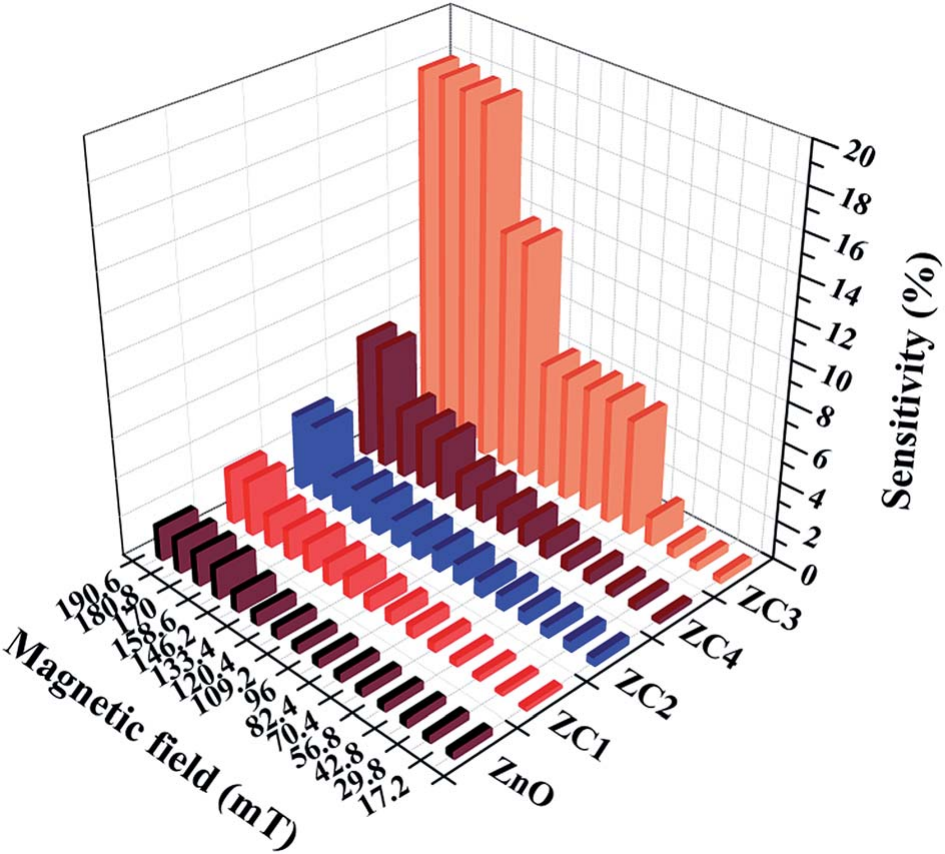
Magnetic sensitivity of the proposed sensor with magnetic field strength ranging from 17.2 mT to 190.6 mT.

Fig. 13 shows the magnetic field sensitivity plot of pristine and Co doped ZnO nanorods in different magnetic field ranging from 17.2 mT to 190.6 mT at an ambient temperature of 28 °C. From Fig. 13, it can be seen that Co doped ZnO nanorods (*x* = 0.05, 0.1, 0.15 and 0.2) show enhanced sensitivity compared to pristine ZnO. Particularly, Co doped ZnO nanorods (*x* = 0.15) exhibit a maximum sensitivity of ∼18% compared with that of other Co doped ZnO nanorods (*x* = 0.05, 0.1 and 0.2). On the basis of the data plotted in Fig. 13, we can summarize that the sensor response increases fairly up to a Co doping of *x* = 0.15, then decreases for higher doping concentrations. It is seen that the materials with higher magnetization possess high sensitivity.

Further, beyond the magnetic field of 180.8 mT, the sensor showed a saturated response. Therefore, the operating range for the proposed magnetic sensor is in the range of 17.2 mT to 180.8 mT.

**Fig. 14:**
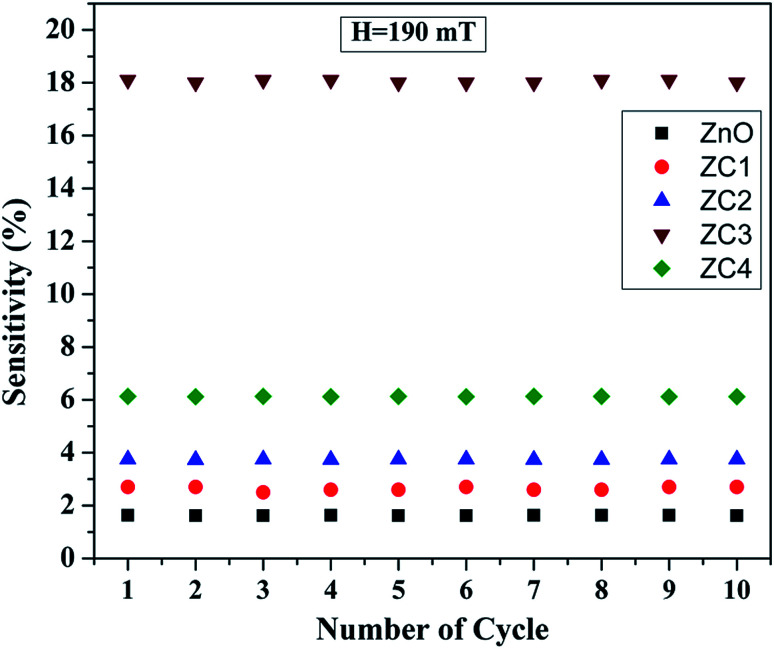
Reproducibility plot of pristine ZnO and Zn_1−*x*_Co_*x*_O nanorods towards magnetic field strength of 190 mT.

In order to study the effect of reproducibility, the sensor was characterized over ten cycles with a magnetic field strength of 190 mT and the experimental results are shown in Fig. 14. It is seen that the sensing response for all cycles are nearly identical with insubstantial sensitivity fluctuations. This clearly depicts that the sensor has good reproducibility.

## 4. Conclusions

A fiber optic magnetic field sensor using Co doped ZnO nanorods (5, 10, 15 and 20 at%) has been proposed and experimentally demonstrated. The analysis confirms that the nanorods are in hexagonal wurtzite structure. Furthermore, the VSM analysis indicates that the Co doped ZnO nanorods exhibit weak ferromagnetism at lower doping levels (ZC1 and ZC2) and paramagnetism at higher doping levels (ZC3 and ZC4). Experimental results show that the sensor has an operating magnetic field range from 17.2 mT to 180.8 mT and revealed the maximum sensitivity of ∼18% for Co doped ZnO nanorods (*x* = 0.15) which shows higher value of magnetization. The proposed magnetic field sensor will serve as a better platform for the development of fiber optic magnetic field sensors as better replacements for current Hall effect based sensors.

## Conflicts of interest

There are no conflicts to declare.

## Supplementary Material
